# Effect of Water Condensate on Corrosion of Wires in Ungrouted Ducts

**DOI:** 10.3390/ma14247765

**Published:** 2021-12-15

**Authors:** Radoslav Ponechal, Peter Koteš, Daniela Michálková, Jakub Kraľovanec, František Bahleda

**Affiliations:** 1 Department of Building Engineering and Urban Planning, Faculty of Civil Engineering, University of Zilina, Univerzitna 8215/1, 010 26 Zilina, Slovakia; radoslav.ponechal@uniza.sk (R.P.); daniela.michalkova@uniza.sk (D.M.); 2 Department of Structures and Bridges, Faculty of Civil Engineering, University of Zilina, Univerzitna 8215/1, 010 26 Zilina, Slovakia; jakub.kralovanec@uniza.sk; 3 Laboratory of Civil Engineering, Faculty of Civil Engineering, University of Zilina, Univerzitna 8215/1, 010 26 Zilina, Slovakia; frantisek.bahleda@uniza.sk

**Keywords:** post-tensioned bridge, prestressing steel, corrosion, assessment, humidity, water-condensate, simulation

## Abstract

In the case of existing prestressed concrete structures, information about the actual state of prestressing is an important basis for determining their load-carrying capacity, as well as remaining service lifetime. This is even more important in the case of existing prestressed concrete bridges, which are exposed to a more aggressive environment than the other prestressed concrete structures. The level of prestressing is affected and reduced by prestress losses at a given time. In calculating the internal forces and stresses, required for the assessment of the Ultimate Limit State and the Serviceability Limit State, it is necessary to know not only the prestressing level but also the cross-sectional area of the prestressing steel (wire, strand or cable), which can change in time due to corrosion. In practice, in the case of the pre-tensioned concrete members, it has often happened in the past that cable ducts have been grouted only partially, or not at all, due to poor grouting technology. Experts did not realize what this could cause in the future—the penetration of water with aggressive agents directly into the cable duct and consequently corrosion of the prestressing steel, which means not increased protection of the steel, but rather acceleration of degradation. On the other hand, in many cases, corrosion also occurs in ducts that are not grouted and no water has entered them. This paper deals with this phenomenon—the formation of corrosion of prestressing steel in cable ducts in ungrouted ducts due to moisture. This problem was investigated experimentally and numerically in the simulation program ESP-r. Experimental measurements and numerical simulations have shown that the water vapor condenses in the cable ducts, which can subsequently cause corrosion of the prestressing steel.

## 1. Introduction

Deterioration of structural materials is an inevitable and challenging fact in the field of engineering. Consequently, we are forced to deal with it from the moment of design and construction of structural members. One particular form of deterioration—corrosion of prestressing steel—affects the load-carrying capacity and remaining service lifetime of structures [1,2,3,4,5,6]. This kind of deterioration is difficult to detect through the regular inspection and belongs among the most critical types of damage in prestressing tendons (wires, strands, or cables) [7,8,9,10,11]. Corrosion can be defined as an electrochemical phenomenon, in which steel essentially returns to its original form—ore—by creating iron oxides on its surfaces [12]. It means that the corrosion is physicochemical reaction between a metal and the environment. Corrosion of metals occurs spontaneously because metal (steel) tends to get into a thermodynamically stable state in which it is found in nature [13]. Ultimately, all engineering materials will return to their original state, the forms which are found in nature [14]. The speed of the propagation of reinforcement corrosion is influenced by various endogenous and exogenous factors. On one hand, endogenous factors include chemical and physical inhomogeneity of the surface, the composition of the metal and surface treatment. On the other hand, exogenous factors consist of components such as air pollution, relative humidity, or climate—temperature, precipitation, wind [15]. Four fundamental components are important for corrosion to take place—an anodic reaction, a cathodic reaction, an electrolyte and an electronic path [14].

**Figure 1:**
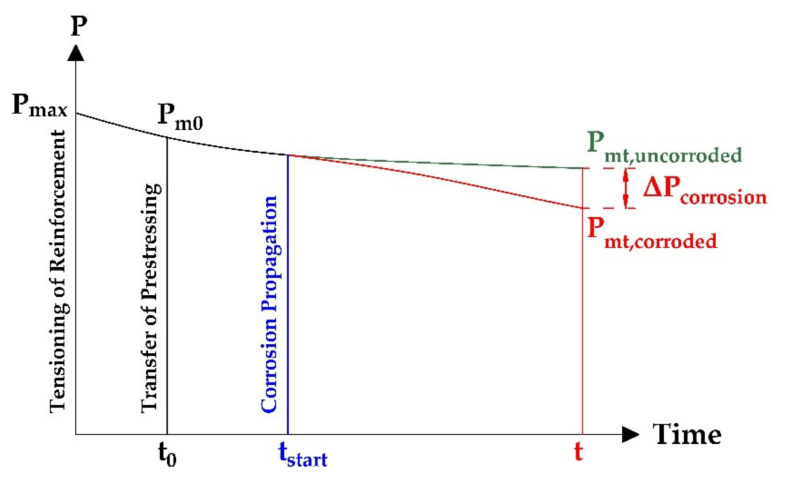
Influence of prestressing steel corrosion on change of prestressing force.

In terms of material deterioration, the prestressed concrete (PC) structures are not an exception. In the case of the post-tensioned structures, the prestressing force is applied by jacking steel tendons against an already-cast member, so the prestressing is transferred through built-in steel anchorages. Furthermore, if the area between the prestressing steel and the duct is injected with cement grout, the prestressing is transferred also through the bond between the steel strands or wires and concrete. This cement grout with plastic or steel duct also provides efficient protection of strands (or wires) from corrosion [16]. Steel cable ducts were used mainly in the past. Nowadays, plastic cable ducts are commonly used. Similarly, in the case of reinforced concrete (RC) structures, the reinforcement is protected by surrounding concrete [17]. Unfortunately, in practice, we can find existing post-tensioned bridges with only partially grouted or even ungrouted ducts. Consequently, prestressing steel located in these ducts is prone to corrosion as the surrounding protective layer of cement grout is missing. Once corrosion is discovered in a prestressing tendon, it is a challenging task to quantify the degree of corrosion and its location through the entire length of the tendon [18]. Corrosion losses cause a decrease in the cross-sectional area of the prestressing steel (wires, strands or cables), and thus lead to additional losses of prestressing force, which negatively affects the load-bearing capacity in Ultimate Limit State (ULS) and the reliability and remaining service lifetime of the prestressed concrete bridge [19,20,21,22,23], see Figure 1. These additional prestress losses are not considered in standard design according to Eurocodes, so their monitoring and subsequent determination is a very important part of the structural assessment. As a result of insufficient maintenance, corrosion-induced failure may cause an unexpected collapse of a prestressed concrete bridge (or structure) [24,25].

Evaluation of corrosion losses of prestressing steel in ungrouted ducts was investigated in scientific work [14]. This research was performed on prestressing strands in unstressed conditions (without prestressing), whereas the prestressing strands were placed in isolated ungrouted ducts for a certain amount of time (1 week, 2 weeks, 4 weeks, 8 weeks and 9 months). Moreover, the humidity during the experimental investigation was ranging from dry to wet. However, the experimental program was focused on observation of corrosion of prestressing steel during the so-called—ungrouted period. This period expresses the time when strands are left without any protection, for example for the duration of the erection of a post-tensioned bridge. On the contrary, the presented article is focused on simulations performed in a long-term period of service life in which the ducts are ungrouted permanently.

This paper deals with the problem of corrosion of prestressing reinforcement in long-term ungrouted cable ducts on real bridge structures. The aim is to present that even if water does not get directly into the ungrouted cable duct, corrosion of the prestressing steel can occur due to natural moisture in the duct.

## 2. Description of a Studied Bridge

**Figure 2:**
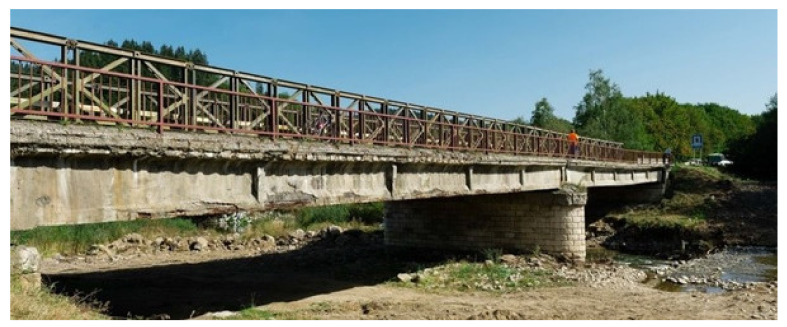
View on studied bridge structure in Podbiel.

The pivotal object of the presented study is one of the post-tensioned bridges in Podbiel (north part of Slovakia) from the first generation of prestressed concrete bridges which were built in former Czechoslovakia in the middle of the previous century (in 1956). The investigated bridge is presented in Figure 2.

**Figure 3:**
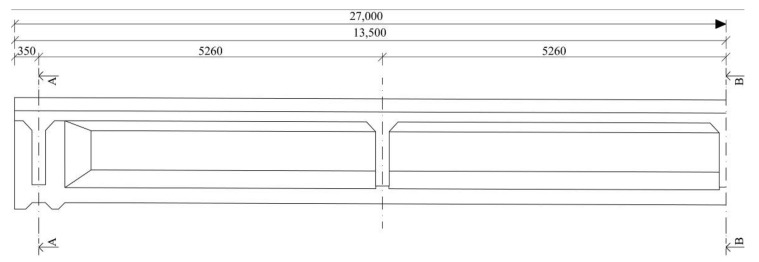
Longitudinal layout of post-tensioned beam of investigated bridge.

**Figure 4:**
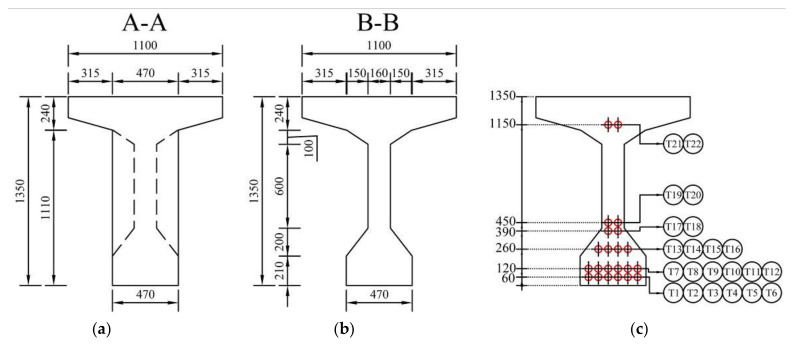
Cross-section of the beam: at end A-A (**a**); mid-span B-B (**b**); arrangement of tendons (**c**).

At the time of erecting, similar bridges consisting of precast post-tensioned girders were built and they deal with the same problems—corrosion of prestressing steel due to ungrouted cable ducts. This structure was chosen as an example because the diagnostic survey provided reliable information about the state of grout in ducts which are crucial for the presented analysis. The bridge in question was located in northern Slovakia on an international road leading to Poland. At the time of the diagnostic survey, this bridge was in service for almost 60 years. Edge supports of the bridge were provided by abutments and middle support was represented by a pillar. The span No. 1 was crossing the river terrace, whereas the span No. 2 was crossing an adjacent creek. The analyzed bridge consisted of two simply supported spans with an identical effective length of 26.650 m. The structure consisted of ten individual precast post-tensioned beams with a spacing of approximately 1140 mm. Their height was 1350 mm. The overall width of the structure was 11,800 mm. In the 1950s and 1960s, the deck of this type of bridge was normally composed of a monolithic concrete slab, which was cast on the upper edge of the precast beams. Nevertheless, the rigid connection between the precast beams and the monolithic concrete slab was not ensured since the slab was designed only as the base of the bridge pavement to ensure a transverse slope. Thus, the concrete slab was considered as ballast and did not contribute to the load-carrying capacity of the investigated bridge. The transversal connection between the beams was established using the transversal prestressing which was typically located in their upper flanges (in this case the spacing was 320 mm) and transversal diaphragms (with an axial distance of 5240 mm). Longitudinal prestressing was transferred into the precast concrete girders by twenty-two tendons consisting of twelve patented wires with a diameter of 4.5 mm. Prestressing wires were placed in a cable duct of a diameter of 50 mm. Cable ducts were identified as metal tubes [26,27,28]. Detailed drawings of the bridge used in the analysis are shown in Figure 3 and Figure 4.

**Figure 5:**
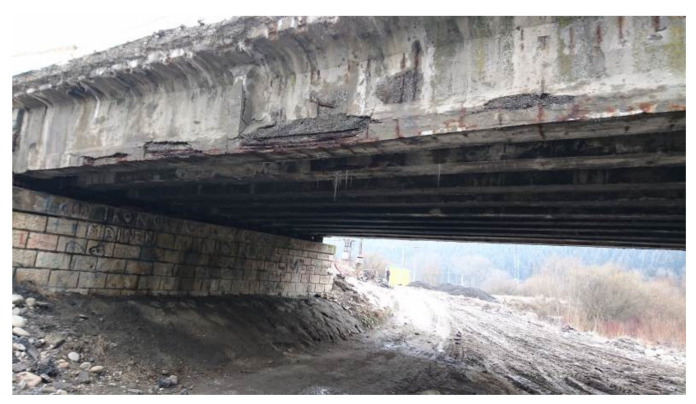
Bridge and its state prior to demolition (span No. 1).

**Figure 6:**
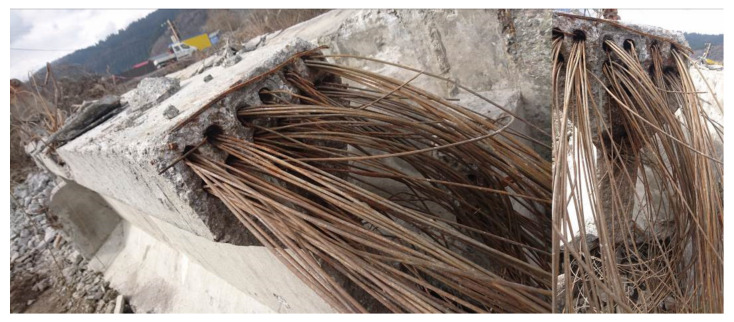
View on ducts after the bridge demolition (unfilled ducts and visible corrosion).

The investigated bridge was in a bad state at first sight, so it could be assumed that regular inspections were neglected. The view on the bridge prior to its demolition can be seen in Figure 5. The results of performed inspection required immediate intervention in form of bridge closure for traffic. Completed tests indicated severe propagation of corrosion of prestressing steel (wires and anchorages). This fact dangerously contributed to additional prestress losses and the reduction of the load-carrying capacity of the bridge in question. The state of cement grout in ducts was investigated after the demolition of the bridge, see Figure 6. The conclusions of this investigation were alarming, as the ducts were empty or only partially filled with cement grout. Consequently, the prestressing was unbonded and prestressing steel was exposed to a significant risk of corrosion.

A presented study should help to understand the reasons for such severe corrosion of prestressing wires in ungrouted ducts of post-tensioned bridges and describe the conditions which negatively contributes to its extensive propagation like in the case of an investigated bridge. Additionally, the performed analysis could provide important information for the operators of post-tensioned concrete bridges from the first generation which could be endangered by analogous issues. 

## 3. Vapor Condensation Theory

Condensation on the surface of the air cavity can occur when the surface temperature of the material surrounding the air cavity is lower than the dew point temperature. If the air cools near a cold surface, its relative humidity in this area can increase significantly. If it cools too much, condensation will form, liquefying excess water vapor. The determination of a dew point temperature D_p_ depends on the temperature T (°C) and the relative humidity RH (%) according to the formula [29]:

(1) $$D_{p}(T,RH)=\frac{243.12\cdot \left(\ln\left(\frac{RH}{100}\right)+\frac{17.62\cdot T}{243.12+T}\right)}{17.62-\left(\ln\left(\frac{RH}{100}\right)+\frac{17.62\cdot T}{243.12+T}\right)}$$

This equation is a commonly used approximation in the range of −45 to +60 °C. For instance, the dew point temperature is 0.9 °C for relative humidity RH = 75% and a temperature of 5 °C. The problem of water vapor condensation in closed cavities is often occurring phenomenon in building roofs and facades. If it is covered with metal sheeting (metal is a good heat conductor), its surface can cool very sharply and condensation can form on its surface [30,31]. The same problem occurs in the case of a post-tensioned bridge with steel cable ducts. 

## 4. Experiment with Relative Humidity of Air in Cavity

The relative humidity rate in the cavity is the most significant for the cavity with the most negligible concrete wall/slab thickness. Due to the unavailability of information on humidity conditions in closed cavities of concrete elements (ducts), it was necessary to perform an experiment to describe this phenomenon. The fundamental question was whether it was possible to achieve such humidity in the cavity that it began to condense and subsequently cause corrosion of the prestressing steel. The results from the experimental program will be compared to the simulations (see Section 5). In the prepared experimental measurement, two procedures were chosen to achieve a change in humidity in the cavity (representing the duct). The first was to monitor the humidity change in the cavity under the influence of atmospheric humidity at a constant ambient temperature, which should be a lengthy process of water vapor entering the duct, as the temperature in the cavity and the ambient temperature are the same. The second way to increase the moisture was to place the test specimens in an aqueous medium at the same temperature as the test specimens. The second method is to simulate the inflow of water (flowing water) onto the surface of concrete beams. The water transport through the porous material can also be monitored by simulation if the input data are available [32,33]. The chosen test procedure neglects the influence of the concrete element size with its absorbency and the uneven distribution of concrete around the channel. The aim was to verify whether the humidity in the cavity could reach the value used in the simulations (Section 5).

**Figure 7:**
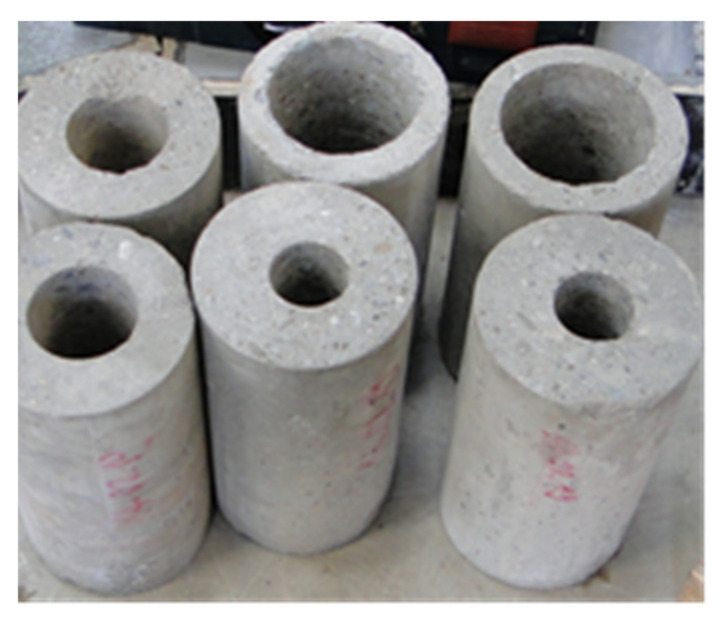
Concrete specimens with varying cover thickness.

Samples of dimensions of 150 × 300 mm (concrete cylinders) served to describe the change in humidity in the duct with no airflow. The cavity in the specimen originated in the longitudinal drilling of holes of different diameters. The holes of diameter 107 mm in specimens with denotation “C21”, holes with a diameter of 72 mm in specimens with denotation “C40” and specimens with denotation “C50” with the holes of diameter 51 mm were created (see Figure 7). This marking represents the concrete cover layer thickness “c” (for instance, denotation C21 means the concrete cover c = 21 mm, it is the thickness of the cylinder wall). It simulates the concrete cover layer of the steel duct. The base of the cylinders was modified with a plastic cover so that no moisture could penetrate through them. A miniature multi-sensor module for measuring temperature and humidity from Ahlborn was used in the middle of the cavity. A Pt100 surface temperature sensor from Ahlborn was fitted on the outer surface of the samples.

The concrete cylinders were 27 months old, with a concrete cube strength f_ck,cube_ = 58.1 MPa and an average bulk density of 2295 kg/m^3^. The maximum aggregate size was 16 mm, and the cement CEM II BS 32.5/R was used.

### 4.1. Atmospheric Humidity—Dry Samples

**Figure 8:**
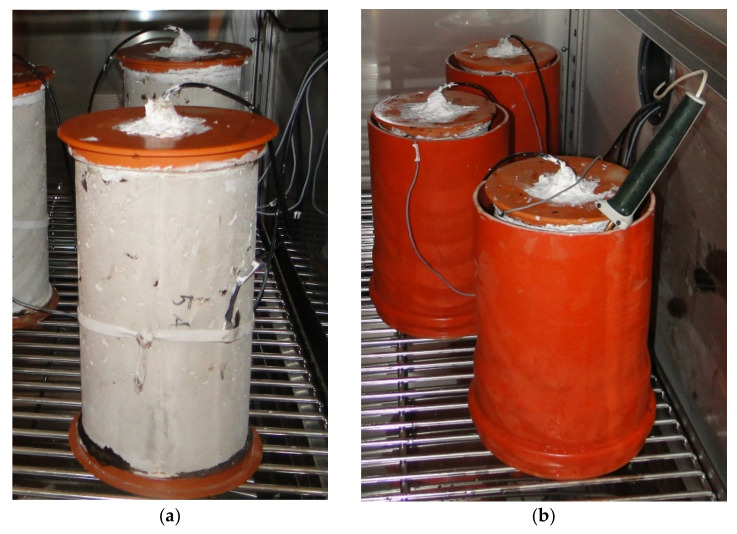
Samples in the climatic chamber: (**a**) atmospheric humidity, (**b**) water storage.

The temperature for the drying of samples was 110 °C (230 °F). After the drying process, they moved to an air-conditioning chamber. The ambient temperature in the air conditioning chamber was the lowest (8 °C), at which it can maintain a humidity of 90% for a long time. The temperature and humidity were independent controlled in the inside space of the chamber. To determine the water absorption, it was necessary to determine the weight of the samples, measured at selected time intervals. After reaching humidity of 75% inside the specimen denoted as C21, the experimental test was terminated. The experiment aimed to determine whether and for how long the moisture in the cavity will reach 75% under constant temperature conditions under the action of atmospheric humidity and what effect the concrete cover thickness of the sample has (Figure 8a). The same samples were used in the second following procedure, as well. 

### 4.2. Wetted Sample—“Flowing Water”

Additionally, we dried identical samples at 110 °C (230 °F). After tempering to 8 °C (46.4 °F), the specimen moved in an 8 °C water storage (water bath). They have been placed in a climatic chamber in the water bath, while maintaining an ambient and water temperature of 8 °C. After reaching the desired humidity in the cavity, we measured the weight of samples to determine the water absorption (Figure 8b).

### 4.3. Results of Experimental Measurements

**Figure 9:**
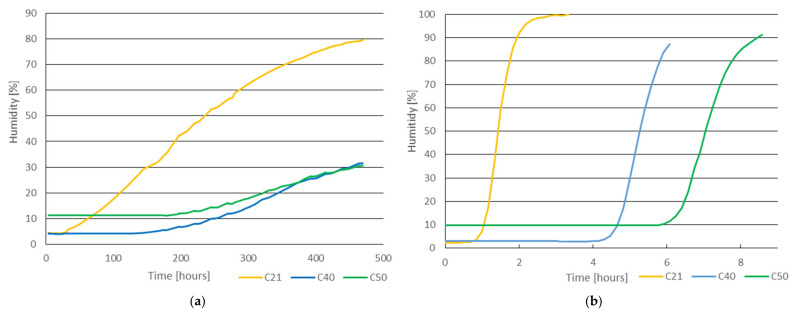
Relative air humidity dependence on cavity when (**a**) exposed to atmospheric humidity; (**b**) placed in water storage.

Figure 9a shows the humidity dependence on the cavity of a concrete sample when exposed to atmospheric humidity and Figure 9b when placed in a water storage (aqueous environment). The surface temperature of the specimen, the water and the cavity temperature were practically the same throughout. It follows from the depicted dependences that the humidity in the cavity begins to grow in both environments only after a specific time—this time interval can be called the passive stage. In the passive stage, water (water vapor) penetrates into the sample but does not affect the moisture in the cavity.

Table 1 shows the length of the passive stage and the total time to achieve a 75% change in humidity in the cavity. The water absorption of the concrete was determined after the test.

Under atmospheric humidity, a 75% increase in moisture in the cavity of the C21 sample occurred after 469 h. In the case of a water storage, that time was 103 min. The significant difference in the length of the passive stage between samples C21 and samples C40 and C50, whether under atmospheric humidity or in water storage, is due to the fact that the maximum aggregate grain size of the concrete mix is 16 mm, with a concrete cover thickness of 21 mm in the case of C21.

**Figure 10:**
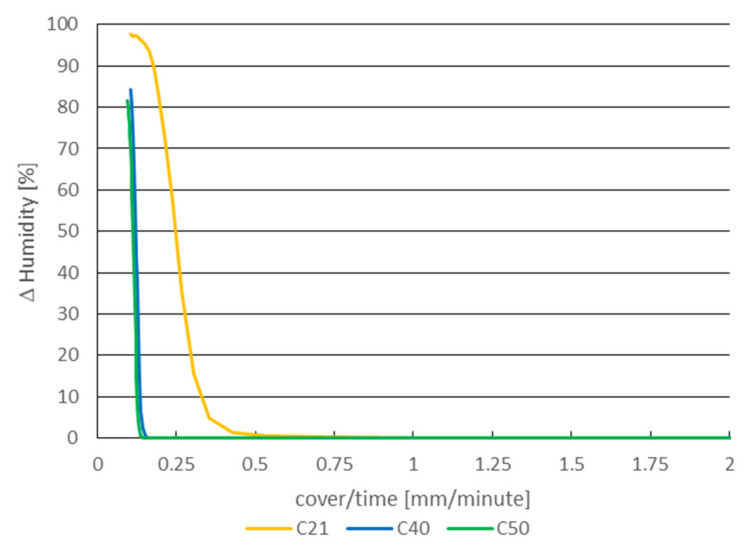
Humidity dependence on ratio of thickness of concrete cover and time—samples placed in water storage.

The following Figure 10 shows the change in humidity depending on the ratio of the thickness of the concrete cover (thickness of cylinder wall) and the time of samples in the water storage, i.e., the rate of change of humidity in the cavity.

The inverse value (ratio) represents the rate of moisture penetration during the passive stage (Figure 10), it is 0.463 mm/min for C21, for C40 it is 0.149 mm/min and for C50 it is 0.138 mm/min. The significant difference between the moisture penetration rate in the case of C21 and in the case of C40 and C50 samples is due to the fact that the maximum size of the aggregate used in the concrete of 16 mm. The depth of the C21 concrete cover is 21 mm and the cavities (holes) were created by diamond drilling, where the aggregate was also cut.

**Figure 11:**
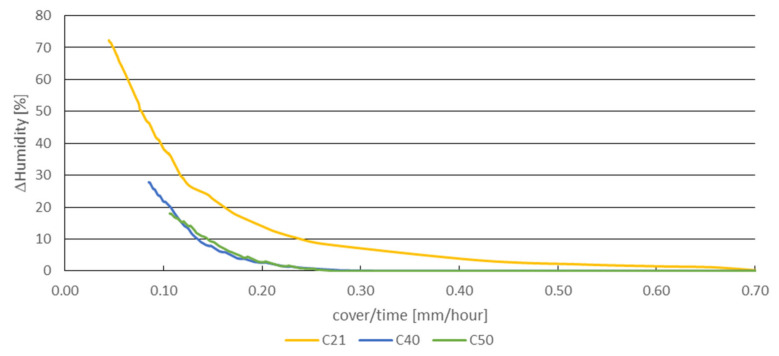
Humidity dependence on ratio of thickness of concrete cover and time—samples placed in atmospheric humidity.

Figure 11 shows the change in humidity depending on the ratio of the thickness of the concrete cover and the time of the samples under the influence of atmospheric humidity.

The rate of moisture penetration during the passive stage (Figure 11) in this case is equal to 0.677 mm/h for C21, for C40 it is 0.240 mm/h and for C50 it is 0.236 mm/h.

## 5. Dynamic Thermal Simulations

Whether the water vapor would condense on the surface of the duct is determined by its current surface temperature. It depends on the external climatic conditions (especially the dry-bulb air temperature and solar radiation) and the heat capacity of the concrete beam. The calculation considers the dynamic behavior performed in the simulation program ESP-r (release 12.0), developed as a research tool [34]. When it comes to simulating the heat capacity, it is one of the most accurate simulation tools using a finite volume approach for solving a set of conservation equations.

**Figure 12:**
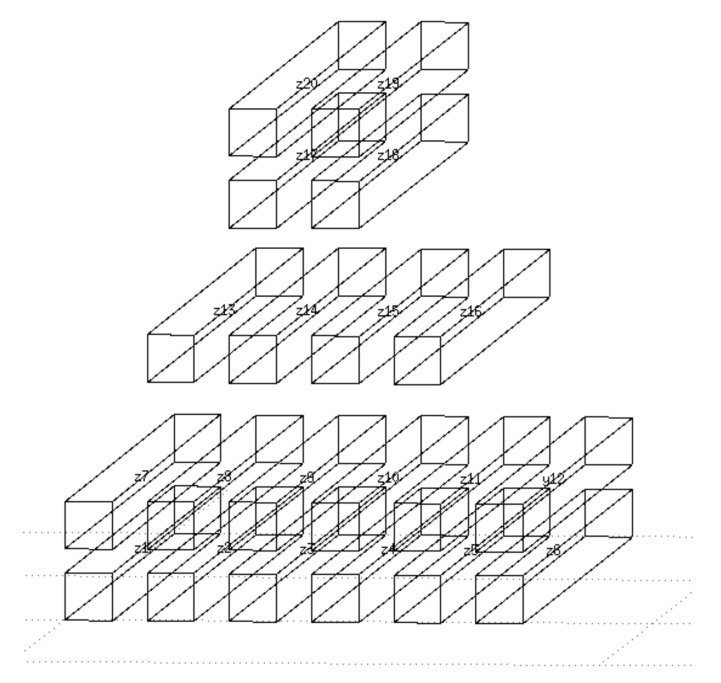
Sketch of a simplified simulation model in ESP-r program (consists of 20 zones representing air cavities (ducts) in a 1 m long sample).

**Figure 13:**
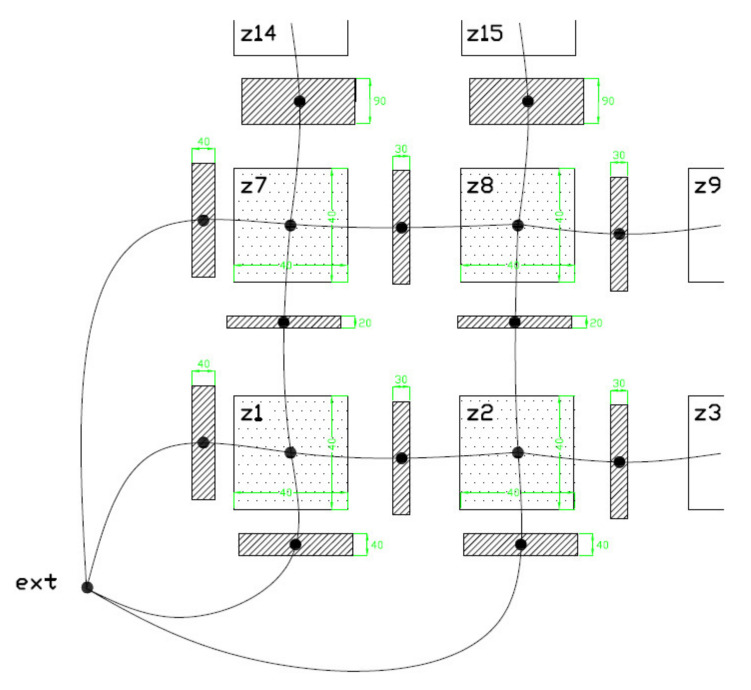
Lower left-hand part of finite volume network in simulation program consists of air cavities and surrounding concrete walls (thin solid line shows simulated thermal bonds).

The simulation model works with a square cross-section of the air cavity (duct) instead of the original circular shape, enabling creating an orthogonal network of these cavities (ducts). The model, representing the cut-out of a one-meter concrete beam, consisted of 20 air cavities (20 zones model) (see Figure 12) with denotation from Z1 to Z20. The concrete walls between the ducts of appropriate thickness interconnected them. The lower left part of this network is shown in Figure 13. The model’s weakness is the lack of a direct connection between the individual concrete walls, which would allow the heat conduction. Without this connection, one wall can be preheated from the sun while the other remains colder. The bulk density of the concrete was considered to be 2400 kg/m^3^, the computing coefficient of thermal conductivity was 1.58 W/m.K, the specific heat capacity was 1020 kg/m^3^, the absorption of the surface for solar radiation is equal to value 0.6. The simulation does not include the moisture transfer. The orientation of the cavities meets the bridge girder in the east-west direction (as original bridge structure). That means that the sunlight in the simulation fell on the left-hand side. The numerical simulation used the reference climate year of the IWEC (International Weather for Energy Calculations) database for Prague. The zone (duct) humidity was a constant value throughout the simulation period. As described in the previously mentioned experiment, it is problematic to determine the relative humidity of the air in the duct. The relative air humidity in the cavity is more affected by the moisture contained in the concrete than in the outside air. For example, the concrete can absorb air humidity by rain and so it can get wet. Water can enter the concrete beam through the leaky road pavement, as well. Therefore, the simulations considered several levels of relative humidity in the cavity: 65%, 70%, 75% and 80%. It is also expectable that the bridges over the river will be less favorable during the year than over the ground (land). 

### 5.1. Condensation Potential on Surface in Cavities

**Figure 14:**
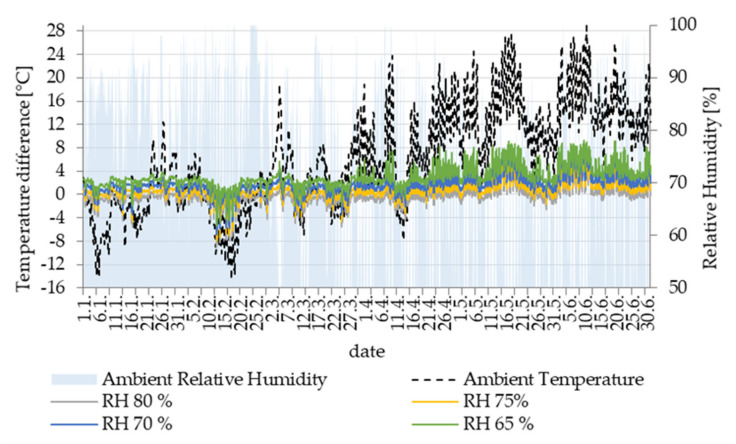
Potential for condensation in Z1 cavity at different considered relative humidity of air in cavity over year.

As will be seen in the next section, the main potential for condensation is in the cavities Z3 and Z4. They are on the bottom edge of the beam, and, in addition, the sunlight is not incident at this side of the concrete beam. However, condensation can occur in several cavities, which can also be seen in Figure 12 and Figure 13. The evaluated potential for condensation depending on the relative humidity in the left-bottom cavity (marked as Z1) is shown in Figure 14. The difference between the surface temperature on the cavity wall and the dew point temperature, determined according to Equation (1), indicates the potential.

**Figure 15:**
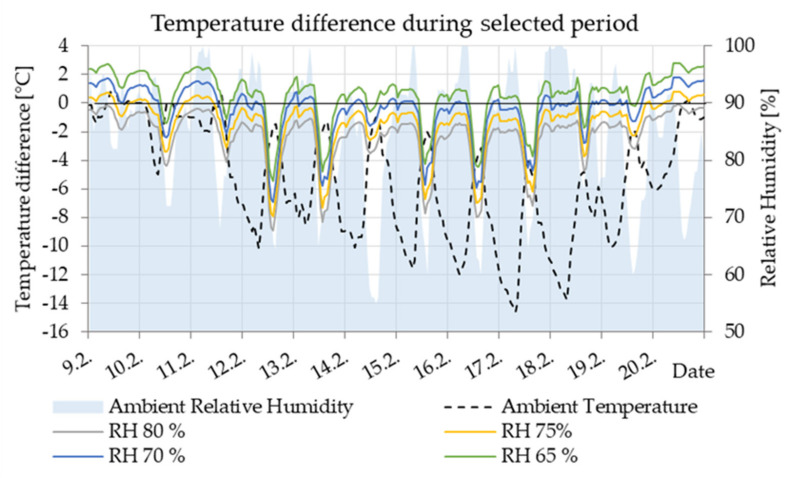
Detail of potential for condensation in Z1 cavity at a different considered relative humidity of air in a very cold period.

According to the course for 75% RH of air in the cavity, condensation can occur, especially in the cold winter season. In general, the lower is the air temperature, the greater the condensation potential is. For this reason, the coldest week from 9–20 February, which occurred in the reference climate year, was selected for further analysis in the other cavities. During that period, the relative humidity of the outside air was high for a long time, fluctuating from 60% to 100% (Figure 15).

### 5.2. Simulation Results

#### 5.2.1. Cavity Z1

**Figure 16:**
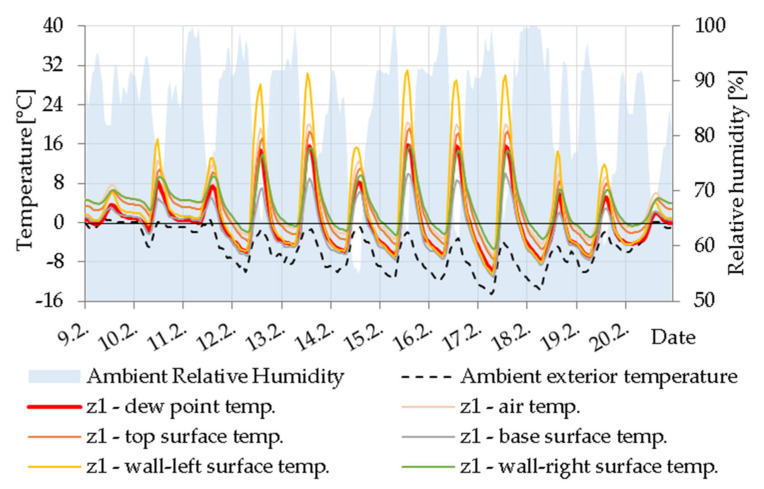
Temperature courses in cavity Z1.

Figure 16 shows a significant difference in surface temperature and dew point temperature practically throughout the day. The air in cavity Z1 heats up quickly from the dazzled side of the beam (wall 1), but the lower part and the inner part of the beam remains cool. The bottom wall (in this case, it is a concrete cover layer) is slightly colder than in reality due to the lack of connection to the wall heated by convection in the simulation.

#### 5.2.2. Cavity Z3 

**Figure 17:**
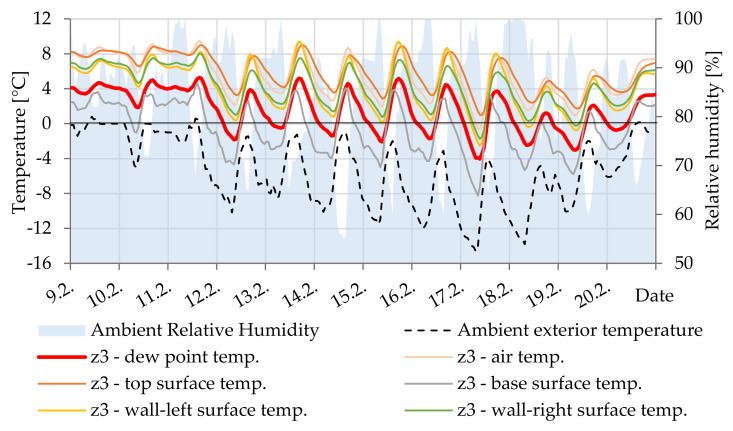
Temperature courses in cavity Z3.

In Figure 17, the difference between the surface temperature and the dew point temperature documents the condensation of water vapor on the lower surface of cavity Z3. By simulation, this surface temperature is always a few degrees lower than the air temperature. At 75% relative humidity in the cavity, this is sufficient to cause condensation.

#### 5.2.3. Cavity Z6

**Figure 18:**
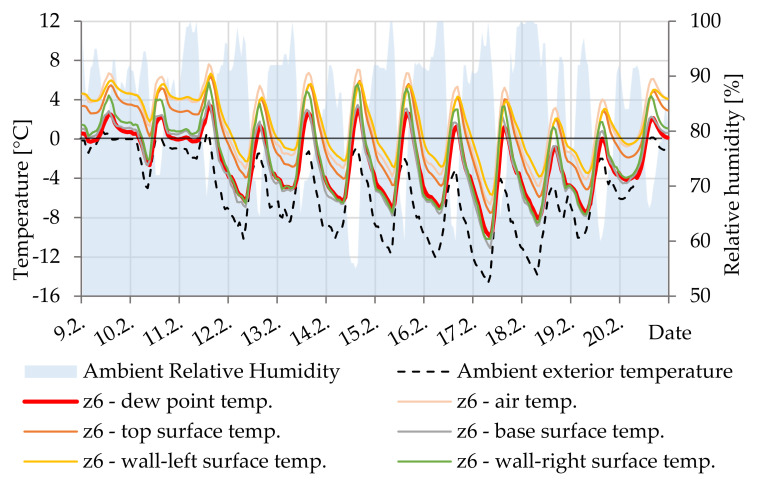
Temperature courses in cavity Z6.

In Figure 18, is shown the difference between the surface temperature and the dew point temperature documenting the possible occurrence of minimal condensation of water vapor on the lower surface of the cavity Z6.

#### 5.2.4. Cavities Z7 and Z13

**Figure 19:**
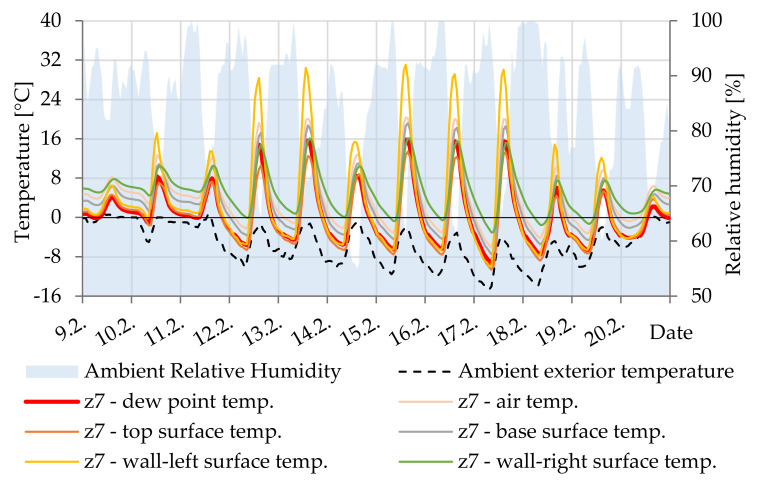
Temperature courses in cavity Z7.

In Figure 19, the difference between the surface temperature and the dew point temperature documents the possible occurrence of minimal condensation of water vapor on the lower surface of the cavity Z7. Inside the beam (surface Z7-base and Z7-wall-right) the temperature is more stable than at the edges (surface Z7-top and Z7-wall-left—“concrete cover”), which are more cooled and heated during the day by the outside environment.

#### 5.2.5. Cavities Z9 and Z14

**Figure 20:**
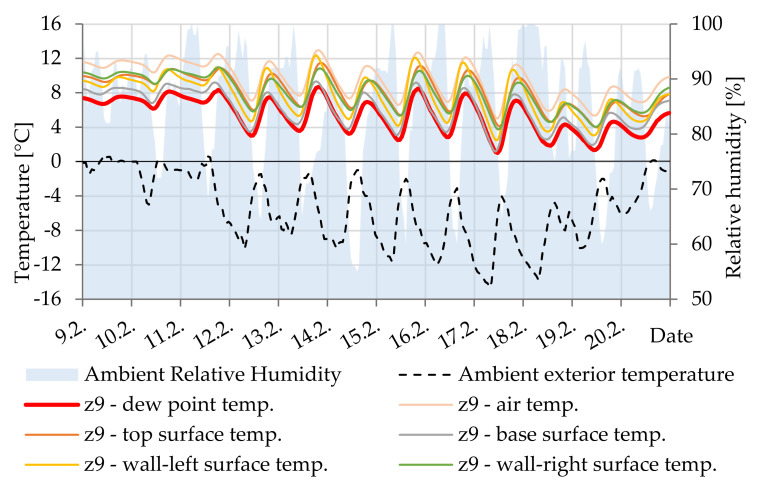
Temperature courses in cavity Z9.

In Figure 20, it is evident that the surface temperature is always higher than the dew point temperature. Due to its storage capacity, the core of the beam maintains at a relatively stable temperature, compared to the outer parts.

#### 5.2.6. Cavities Z12 and Z16

**Figure 21:**
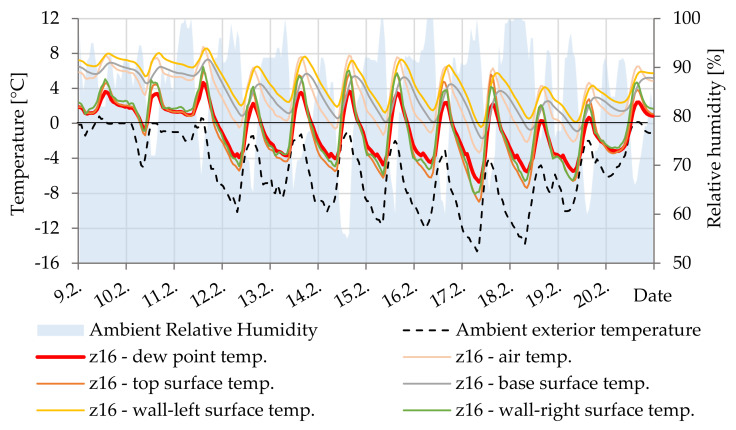
Temperature courses in cavity Z16.

In Figure 21, it is evident that the surface temperature is always higher than the dew point temperature. Due to its storage capacity, the core of the beam maintains at a relatively stable temperature compared to the outer parts.

#### 5.2.7. Cavities Z17, Z18, Z19 and Z20

**Figure 22:**
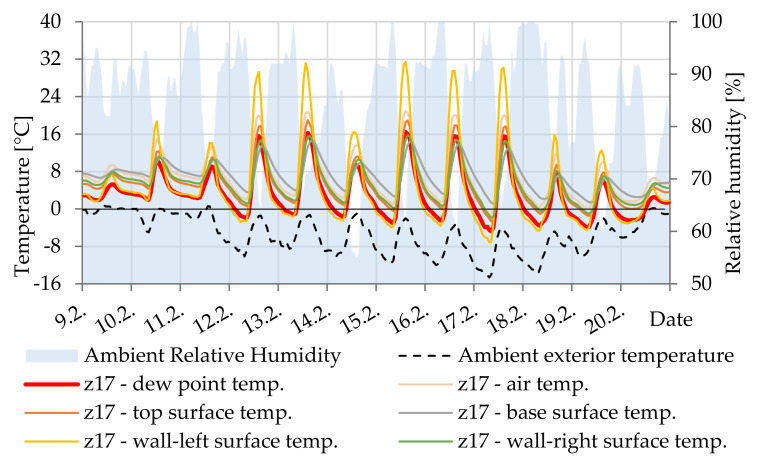
Temperature courses in cavity Z17.

Figure 22 shows that the surface temperature is slightly lower than the dew point temperature. In this case, if condensation also occurs on the concrete surface, it will absorb it. The small amount of condensation does not pose a high risk of corrosion to the steel reinforcement in the cavity.

In addition, through the relatively thin sidewall between the cavity and outside environment the air in the cavity (duct) heats up or cools down rapidly. Still, the massive concrete part of the beam next to the cavity ensures thermal stability. In the long term, if condensation on the surface freezes, it may not melt during the day due to thermal stability. This can also affect the corrosion of the prestressing steel.

## 6. Discussion

Experimental measurements (Section 4) have clearly shown that sufficient moisture (75–80%) can enter the ungrouted cable ducts to cause corrosion of the prestressing steel over time. Moisture can enter the cavities through the concrete material of beam structure (experimentally verified), as well as by flowing through the anchor areas in the case of non-functional insulation (often observed in practice during the real bridge structures diagnostics). 

Experiment in climatic chambers shows that the humidity in the edge cavities will be very strongly dependent on the soaking of the concrete beam by the wind-driven rain. It would be interesting to extend research in this direction and to monitor experimentally, or by simulation, the amount of incident rain, as is done in buildings [35]. 

**Figure 23:**
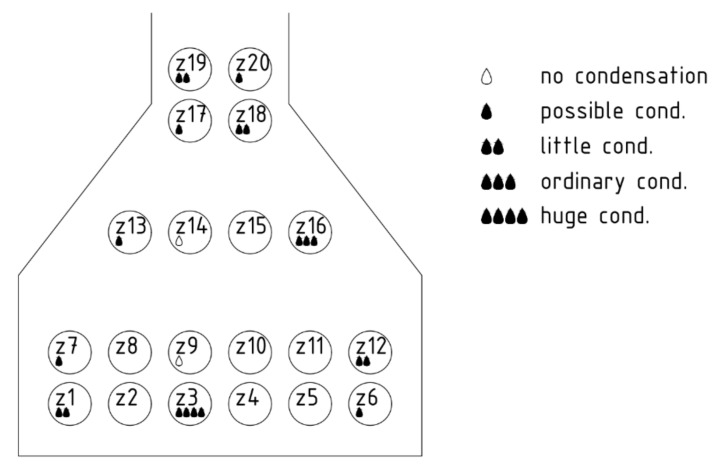
Map of condensation risk from simulation results in selected cavities.

The numerical simulation (Section 5) showed that in the winter period (the period considered during 9–20 February —real temperature measurements) can occur such temperatures on the surface of the cable duct when the air humidity begins to condense. These are mainly cable ducts most exposed to the weather—in the corners and on the underside of the beams (cavities 1 to 6, 12 and 16), see Figure 23. There is also a high probability of condensation in the case of cavities 7 and 13, which could sometimes be heated by sunlight. Conversely, in the cavities in the core of the beam, condensation of moisture is not expected (cavities 9 and 14 and others). The core of the beam does not cool much at night due to accumulations, which has a significant effect on formation of condensation.

Thus, experimental measurements and numerical simulations together proved why in real bridge structures with ungrouted cable ducts (Section 2), an environment suitable for formation of corrosion of prestressing steel is created in the ducts.

## 7. Conclusions

The basic aim of the cable ducts in the post-tensioned prestressed concrete members is the possibility to prestress the concrete element even after stiffening and hardening of the concrete (unlike pre-tensioned prestressed concrete elements). This also makes it possible to trace the position of the cables before concreting, i.e., the possibility of a covered cable (in the ground plan or height). Another important task is to protect the prestressing reinforcement itself against the penetration of degradation factors from the surrounding aggressive environment in which the element is located (mostly bridges). However, it can only perform this function if the cable duct is also well and sufficiently grouted. Today, the plastic cable ducts currently in use perform this task well. However, in the past, metal cable ducts have been used, which could corrode on their own. In the case of post-tensioned prestressed girder bridges, often used in practice in the past (60s to 80s of the last century), it has been proven many times that these cable ducts were not sufficiently grouted—within the diagnoses of bridges from that period it was found that they are either only partially grouted or even not grouted at all.

The presented research shows that in that case, the cable ducts do not protect the prestressing steel against corrosion, but on the contrary, create an environment that can cause corrosion due to moisture condensation without aggressive factors—moisture can get into the cable ducts and water condenses due to temperature, which creates an ideal environment for corrosion of the prestressing steel, which is not protected by the concrete passive layer around the steel as in the case of reinforcement in reinforced concrete structures or prestressing steel in the pre-tensioned prestressed concrete structures.

The research presented in the paper proves that the cause of problems of the post-tensioned prestressed bridges in practice, in which the prestressed reinforcements were not grouted well was corrosion of prestressing steel, even without penetration of aggressive factors to the steel itself, which could subsequently cause sudden failure of elements much earlier than their planned lifetime (approximately after 40 to 60 years of the service lifetime). The failure is usually sudden without showing any previous indications that would point to a possibility of a sudden failure (spalling the covering layer, the formation of primary cracks, large deflections, etc.).

For this reason, in the case of the post-tensioned prestressed bridges, it is necessary to pay increased attention to determining the actual state of grouting of cable ducts and the state of the prestressing reinforcement—whether it is corroded or not.

## Figures and Tables

**Table 1:** Time Course of Humidity and Absorptivity in Cavity.

Samples Notation	Water			Air		
	Passive Stage	by Δ75%	Water Absorption	Passive Stage	by Δ 75%	Water Absorption
	min	min	%	hours	hours	%
C21	48	103	4.35	31	469	0.223
C40	260	344	4.49	167	469 *	0.182 *
C50	361	484	4.62	212	469 *	0.145 *

* Completion of the test together with C21.

## Data Availability

Not applicable.
